# Application of a new MDCKII-MDR1 cell model to measure the extent of drug distribution in vitro at equilibrium for prediction of in vivo unbound brain-to-plasma drug distribution

**DOI:** 10.1186/s12987-023-00495-4

**Published:** 2024-01-25

**Authors:** Kristine Langthaler, Christopher R. Jones, Lasse Saaby, Christoffer Bundgaard, Birger Brodin

**Affiliations:** 1 Translational DMPK, H. Lundbeck A/S, and CNS Drug Delivery and Barrier Modelling, University of Copenhagen, Ottiliavej 9, Valby, 2500 Copenhagen, Denmark; 2 PKPD Modelling & Simulation, H. Lundbeck A/S, Ottiliavej 9, Valby, 2500 Copenhagen, Denmark; 3 Bioneer A/S and affiliated associate professor at CNS Drug Delivery and Barrier Modelling, Universitetsparken 2, 2100 Copenhagen, Denmark; 4 Translational DMPK, H. Lundbeck A/S, Ottiliavej 9, Valby, 2500 Copenhagen, Denmark; 5 CNS Drug Delivery and Barrier Modelling, University of Copenhagen, Universitetsparken 2, 2100 Copenhagen, Denmark

## Abstract

Intro: Reliable estimates of drug uptake from blood to brain parenchyma are crucial in CNS drug discovery and development. While in vivo K_p,uu,brain_ estimates are the gold standard for investigating brain drug disposition, animal usage is a limitation to high throughput application. This study investigates an in vitro model using P-gp expressing MDCKII-MDR1 cells for predicting in vivo brain drug penetration. Methods: In vitro equilibrium distribution studies were conducted in apical and basolateral solutions with high protein content to estimate K_p,brain_ and K_p,uu,brain_ values. The correlation between in vitro and in vivo K_p,brain_ values for a set of compounds was examined. Results: We observed a good correlation between in vitro and in vivo K_p,brain_ values (R^2^ = 0.69, Slope: 1.6), indicating that the in vitro model could predict in vivo drug brain penetration. The ‘unilateral (Uni-L)’ in vitro setup correctly classified 5 out of 5 unrestricted compounds and 3 out of 5 restricted compounds. Possible reasons for the observed disparities for some compounds have been discussed, such as difference in transport areas between in vitro and in vivo settings and effect of pH changes. Conclusion: The in vitro assay setup developed in this study holds promise for predicting in vivo drug brain penetration in CNS drug discovery. The correlation between in vitro and in vivo K_p,brain_ values, underscores that the model may have potential for early-stage screening. With minor refinements, this in vitro approach could reduce the reliance on in vivo experiments, accelerating the pace of CNS drug discovery and promoting a more ethical research approach.

**Supplementary Information:**

The online version contains supplementary material available at 10.1186/s12987-023-00495-4.

## Introduction

The inability of drug compounds to overcome restrictions enforced the blood–brain barrier (BBB) in order to reach their intended brain targets is a major reason for failure in CNS drug development [1, 2].

As such*, *in vitro screening methods are important in early stages of CNS drug discovery due to their cost-effectiveness and the possibility to test a high number of compounds over a relatively short amount of time. In vitro cell monolayer models exhibiting traits of the native brain capillary endothelium are commonly used to estimate transport rates (flux) and corresponding permeabilities (P_app_) of drug compounds. Drug concentrations in plasma and brain are measured in vivo and provide information on both drug uptake rates, but also the concentration of free drug in the brain, when combined with estimates of drug-protein binding in brain and plasma (or through microdialysis). Classical measures of brain drug distribution include K_p,brain_ the ratio of drug concentrations in brain and plasma at equilibrium (or the ratio AUC_brain_/AUC_blood_ at non-equilibrium) and K_p,uu,brain_, the unbound brain-to-unbound plasma ratio at equilibrium, thus giving a measure of the extent of the uptake [3–5].

There is a growing focus on replacing animal experiments with cell systems in the field of drug development. This shift is driven by several factors, including ethical concerns about animal usage in research as well as costs associated with in vivo studies [6–9].

The MDCKII-MDR1 cell line, a canine kidney cell line transfected with human p-glycoprotein, is commonly used as an in vitro model to predict BBB permeation of drug compounds. This cell line is characterized by a low paracellular permeability (mannitol flux in the range of 1–7 × 10^–7^ cm/s [10, 11]) and expression of the human efflux transporter P-gp (ABCB1) in the luminal membrane [12, 13]. Cell monolayers of the MDCKII-MDR1 cell line therefore mimics the BBB in terms of physical tightness and efflux transporter expression. Using this system, measured apparent bi-directional permeabilities and derived efflux ratios (ER) coupled with scaling factors have been employed to quantitatively predict K_p,uu,brain_ [14, 15]. Other in vitro models have been developed to attain improved resemblance with the BBB physiology [16–19]. To this end, Culot et al. utilized a co-culture system involving bovine brain capillary endothelial cells and rat glia cells. The presence of glia cells in the abluminal compartment aimed to replicate the non-specific binding of drug compounds in the brain. While these experiments showcased the prospective value of in vitro K_p,brain_ assessments, they did not explicitly consider factors such as plasma binding or full equilibrium, albeit an estimate of equilibrium was made. Thus, in our current study, we expanded the mindset by incorporating equilibrium and fine-tuning of the protein content.

The extent of brain uptake of a drug is determined by several factors, including the physiochemical properties of the drug, its non-specific binding to proteins, the permeability of the BBB and potential affinity for efflux transporters.

The aim of this work was therefore to explore the possibility of estimating in vivo K_p,brain_ and K_p,uu,brain_ values from in vitro experiments at equilibrium with cell monolayers and protein-containing experimental solutions, using BSA as a surrogate for plasma and brain homogenate. For this purpose, two different experimental protocols were investigated, using unilateral (Uni-L) or bilateral (Bi-L) transport experiments with cell monolayers of the MDCKII-MDR1 cell line on Transwell supports, which included a set of 16 compounds with varying physicochemical properties. Prediction performance of these in vitro approaches were assessed by comparing in vitro derived K_p,brain_ and K_p,uu,brain_ values with in vivo values obtained in a previous study [20]. The Uni-L method accurately categorized all 5 unrestricted compounds, and 3 out of 5 restricted compounds, offering potential for predicting in vivo drug brain penetration in CNS drug discovery.

## Materials and methods

### Chemicals and matrices

The chemicals and reagents, including Altanserin, antipyrine, atenolol, buspirone, cimetidine, citalopram, N-desmethylclozapine, diphenhydramine, doxepin, fluoxetine, gabapentin, indomethacin, metoclopramide, propranolol, risperidone, and Way-100635 were purchased from Sigma Aldrich (St. Louise, Missouri, US). The calibration curves and matrices for the in vitro equilibrium brain-to-plasma distribution experiments, were prepared using BSA purchased from Sigma Aldrich (≥ 98%, St. Louise, Missouri, US, Product no.: A7906 Lot. No.: SLCH8448) and brain homogenate from BioIVT (Westbury, NY, US, Product no. RAT00BRAINMZA, lot no. RAT415237). For the in vitro binding experiments, BSA (purity > 98%) was obtained from Beijing SeaskyBio Technology Co. Ltd. (Beijing, North China, China. Product no.: BSAS, Lot.: 541), and Sprague–Dawley brain homogenate was purchased from BioIVT (Westbury, NY, US, Product no. RAT00BRAINMZA, Lot no.: RAT 472873) and Shanghai Biotechnology Co. Ltd (Shanghai, East China, China. Lot.: 20,221,213). Other chemicals and solvents were of analytical grade and obtained from a commercial supplier.

### In vitro BSA and brain homogenate binding for reference compounds

To convert K_p,brain_ to K_p,uu,brain,_ a quantitative determination of the fraction unbound in Bovine Serum Albumin (BSA), and Göttingen brain homogenate was achieved by equilibrium dialysis using 96-well HTD-dialysis plates (HTD Dialysis LLS, Gales Ferry, CT, UDA, dialysis membranes cut off 12–14 kDa). Previous publications [20, 21] provide detailed description of this methodology. In brief, blank matrices (BSA or brain homogenate) were spiked with compounds to achieve a final nominal concentration of 1 μM (with ≤ 0.5% DMSO) then added (100 μL) to one side of a 96-well HTD-dialysis device. The device was equilibrated against an equivalent volume of 0.1 M phosphate buffered saline for 5 h at 37 ℃, with shaking in an incubator with 5% CO_2_. A 20-fold dilution was applied to both protein matrices (BSA and brain homogenate) from their anticipated in vivo concentration in order to mimic the binding conditions attained in the cell permeability assay set up (see Sect. "Equilibrium distribution studies using MDCKII-MDR1 cells" and "Comparing predicted and experimentally determined free fraction in vitro: evaluating the impact of 35 μM BSA and a 20-fold dilution of brain homogenate."). Meaning that the brain homogenate was diluted in 19 volumes (w/v) of phosphate buffer pH 7.4 and BSA was diluted to a final concentration of 35 μM. The rational for choosing these matrice concentrations is described in Sect. "Equilibrium distribution studies using MDCKII-MDR1 cells".

All compounds were tested in triplicate on one test occasion and displayed ≥ 75% assay recoveries.

The measured unbound fractions in BSA (f_u,BSA_) and brain homogenate (f_u,b_) were calculated according to Eq. 1:

1 $${Measured \,f_u}=\frac{{C_{buffer}}}{{C}_{BSA\,\,or\,Brain}}$$

C refers to the concentration of the compound; and measured f_u_ is the ratio of measured concentrations determined from buffer and diluted matrice samples.

With the intention to minimise the number of required experiments prior to initiating use of the novel in vitro setup, the free fraction of each test compound in each diluted matrices (35 μM BSA and 20-fold diluted brain homogenate) was also predicted using free fraction data measured in 100% matrices (previous published, [20]), and the Eq. 2 by Austin et al. [22]:

2 $$Predicted \,{f}_{u2}=\frac{1}{\frac{{C}_{2}}{{C}_{1}}\left(\frac{1-{f}_{u1}}{{f}_{u2}}\right)+1}$$

f_u2_ represents the predicted free fraction at the concentration of interest (e.g. 35 μM BSA or 20-fold diluted brain homogenate); while f_u1_ corresponds to the initial test protein concentration; C_1_ and C_2_ are the first and second nominal matrice concentrations (with values C_1_ = 1 and C_2_ = 0.05, given a dilution factor of 20). The equation is utilized in a novel manner to its original use for determining non-specific binding to liver microsomes [22].

### Bidirectional transport experiment using MDCKII-MDR1 cells

MDCKII-MDR1 cells transfected with human MDR1, were obtained from the Netherlands Cancer Institute. The cells were maintained in α-MEM containing 10% FBS (Corning, Product no.: 35–081-CV), 100 ug/mL penicillin-G, 100 ug/mL streptomycin, 1% non-essential amino acid and cultured under 5% CO_2_ at 37 ℃ and 95% relative humidity. Cells were grown in culture flasks to 80–90% confluency then seeded onto polyethylene membranes (1.0 µm Pore Translucent PET Membrane. Product no.:351131) in a 96-well insert system at a density of 2.3 × 10^5^ cells/cm^2^ (Falcon, HTS 96 Square Well, Angled Bottom, Plate with Lid. Product no.: 353925). The cells were allowed to grow for 4–7 days to achieve a confluent cell monolayer, as assessed by microscopy and immunostaining of junctional proteins.

Permeability assessment in MDCKII-MDR1 cells was conducted in three replicates in a single occasion. Transport buffer consisted of 1% BSA in HBSS with 10 mM HEPES (pH 7.40 ± 0.05). The final test concentration of compounds was 0.5 μM, control compounds were 2 μM for fenoterol and metoprolol (low and high permeability markers) and 10 μM for digoxin (a P-gp substrate) (final DMSO concentration was 0.6% in both chambers). The experiment was carried out in both apical-to-basolateral (AtoB) and basolateral-to-apical (BtoA) directions for 60 min at 37 ℃ (with 5% CO_2_, and saturated humidity).

All compounds were tested in triplicate on one test occasion. Test compounds were loaded onto either the apical side (75 μL) or basolateral side (275 μL) of the cells, with transport buffer on the opposing side of the cells (e.g. apical 50 μL or basolateral 250 μL). The initial donor concentration (25 μL) was sampled 30 s after drug compounds were loaded onto the plate, resulting in a final incubation volume of 50 μL apical and 250 μL basolateral. At the end of the incubation period, samples (75 μL) were taken from both sides. Donor samples were diluted and mixed with transport buffer (50 μL) before quenching. All other samples were quenched directly with acetonitrile containing internal analytical standards tolbutamide and labetalol (125 μL). The samples were centrifuged and analysed by LC–MS/MS (refer to Sect. "Analysis of compounds in biological matrices" for more details).

Mass balance (%-recovery) of compounds were determined with use of Eq. 3:

3 $$Recovery(\%)=\frac{\left[\left({V}_{r}\times {C}_{r}\right)+\left({V}_{d}\times {C}_{d}\right)+\left({V}_{c}\times {C}_{c}\right)\right]}{\left({V}_{d}\times {C}_{0}\right)}\times 100 $$

where V_d_ and V_r_ are volumes in the donor and receiver chambers, respectively (50 μL apical and 250 μL basolateral); C_0_ is the initial concentration in the donor chamber; C_d_ and C_r_ are the final concentrations of transport compound in donor and receiver chambers, respectively. C_c_ is the compound concentration in the cell lysate solution. V_c_ is the volume of insert well (50 μL). The %-recovery of each compound was calculated both with and without consideration of the amount of compound associated with the filter and cell monolayer (including or excluding the cell lysate part (V_c_ x V_d_) of Eq. 3). All compounds exhibited assay recoveries of ≥ 85% when accounting for the amount associated with the filter and cell monolayer, and ≥ 65% when excluding these contributions (despite compounds doxepin and fluoxetine showing ~ 50% in the AtoB direction).

The apparent permeability coefficient (P_app_) and efflux ratio (ER) were calculated using Eqs. 4 and 5:

4 $${P}_{app}=\left(\frac{d{C}_{r}}{dt}\right)\times \frac{V_r}{(A\times {C}_{0})}$$

5 $$ER=\frac{{P}_{app, AtoB}}{{P}_{app, BtoA}}$$

where dC_r_/dt is the concentration of compound in the receiver chamber as a function of time (µM/s); V_r_ is the solution volume in the receiver chamber (50 μL on the apical side, 250 μL on the basolateral side); A is the surface area of the cell monolayer (0.0804 cm^2^); C_0_ is the initial concentration in the donor compartment; P_app,AtoB_ and P_app,BtoA_ refer to the apparent permeabilities in the respective directions.

Compound permeability was classified as low when P_app_ < 1, moderate when P_app_ ranged from 1 to 10, and high when P_app_ > 10. The ER was employed to classify compounds as likely P-gp substrates when ER > 2, possible substrates when ER > 1.5, and unlikely substrates when ER < 1.5.

### Equilibrium distribution studies using MDCKII-MDR1 cells

MDCKII-MDR1 cells transfected with human MDR1, were obtained from the Netherlands Cancer Institute. The cells were stored frozen in DMEM (StableCell^™^ DMEM -high glucose. Sigma Aldrich, St. Louise, Missouri, US, Product no.: D0819) containing 15% FBS (Gibco, Thermo Fisher, Walthm, Massachusetts, US. Product no.: 10270–106) and 5% DMSO, and cultured in cell media: DMEM supplemented with 10% FBS, 1% non-essential amino acids (Sigma Aldrich, St. Louise, Missouri, US, Product no.: M7145), 1% l-glutamine (Sigma Aldrich, St. Louise, Missouri, US. Product no.: G7513), and 1% Penicillin/streptomycin (Pen/Strep 10 mg/mL, Sigma Aldrich, St. Louise, Missouri, US. Product no.: P0781), under 5% CO_2_ at 37 ℃ and 95% relative humidity. Cells were grown in culture flasks to 90% confluency then seeded onto polycarbonate membranes in 12-well insert systems at a density of 1.9 × 10^5^ cells/cm^2^ (Corning Inc., Corning, New York, US. Product no.: CLS3401. 0.4 μm pore size, an area of 1.12 cm^2^). The cells were allowed to grow for 2–3 days to establish confluent cell monolayers determined visually.

On the day before the experiment naïve rat brains were homogenized in 3 volumes of cell media (se constituents above) (1 + 3, w/v) using focused acoustic ultrasonication (Covaris E220x, Covaris Inc., Woburn, MA) for 3.5 min at a temperature of 7–10 ℃ (using intensifier, duty factor 50, peak incidence power: 500 W, 1000 cycles per burst, average power 250, vertical sweep; range 5 mm, velocity 10 mm/s) and stored at – 80 ℃ until use. Utilizing rat brains as a substitute for minipig brain homogenate was based on literature indicating a lack of species differences in f_u_ binding [23, 20].

On the day of experiment, individual compound stock solutions (4 mM) were prepared using DMSO, except for antipyrine and gabapentin, which were dissolved in water, and risperidone, diphenhydramine, buspirone and indomethacin which were dissolved in methanol. The stock solutions were combined in cassettes containing 3–4 compounds and diluted using cell media to concentrations of 100 μM (solvent conc. 30%). The cassettes were further diluted 100 × in the appropriate diluted matrice (either 20-fold brain homogenate or 35 μM BSA in cell media). The matrices were pH adjusted to pH 7.5 using pH-indicator strips (Supelco, Sigma Aldrich, St. Louise, Missouri, US. Product no. 1.09533). The final compound incubation concentration was 1 μM, with a solvent concentration of 0.3% in both chambers. Initial donor samples (100 μL) were taken prior to loading into Transwells.

The experiment involved spiking compounds to both sides of the membrane, or exclusively on the apical side (BSA), with a 500 μL load on the apical side, and 1000 μL load on the basolateral side (the rational for doing this is provided later in this section). The plates were incubated for 29 h at 37 ℃ (with 5% CO_2_, and saturated humidity). Samples were collected from both chambers at specified time-points (7.5, 20, 24, and 29 h) with 40 μL taken from the apical side and 80 μL taken from the basolateral side. To ensure equilibrium and minimize the number of samples, only two samples were taken per well. All samples were matrice matched (1:1 (v/v), dilution factor of 2) using the opposing matrice, in order that a single standard calibration line could be run for each cassette from matrice matched samples spiked with known compound concentrations. The dilution factor was accounted for in the calculations. Each compound was tested in in triplicate, with six wells (three wells per two time points) on three test occasions.

Measuring the transendothelial electrical resistance (TEER) (Word Precision Instruments, Sarasota, Florida, US. EVOMX, Lot. No.: 95643 A04J) of the cell monolayers before and after the experiments with a “chopstick”-electrode (Word Precision Instruments, Sarasota, Florida, US. STX2 Electrode, Lot. No.: 0103A) served to verify the integrity and permeability of the monolayer.

*Preliminary experiments* were conducted to determine the appropriate incubation settings, focusing on striking a balance between attaining an in vivo-like milieu and maintaining acceptable cell viability throughout the duration of the incubation period. The initial matrices, Göttingen minipig plasma, and rat brain homogenate, both diluted 5 times with water, posed challenges to MDCKII-MDR1 cell viability. This was due to plasma coagulation, the impact of the brain homogenate, and nutrient deficiencies. To address these issues, plasma was replaced with 700 μM BSA [24], and further dilutions were tested using cell media (5-,20, 50- and 100-fold dilutions). 20-fold dilutions were identified as optimal for both BSA (35 μM) and brain homogenate (brain:cell media (1:19)). Equivalent dilutions were selected for both matrices to maintain a consistent ratio of binding sites between the brain and plasma, mirroring the in vivo. From the preliminary experiments, it was observed that some compounds faced challenges to achieving equilibrium within the set incubation time (29 h). To address this, two methods were used: ‘Uni-L’ whereby compounds were only added to the albumin side (mimicking the blood side, in vivo-like conditions), and ‘Bi-L’ whereby compounds were added to both sides (e.g. BSA and brain sides) to accelerate compound equilibrium. Compounds were mixed with the relevant matrices before loading onto cells to account for viscosity. The 29 h incubation time ensured cell viability and maximized potential of achieving equilibrium for the diverse compound set. To avoid interrupting the equilibrium process, only one sample was taken before the final second sample, requiring the use of 6 wells to obtain triplicate data at 4 different time points. Preliminary results were not included in this work (however, a preliminary experiment on matrice dilution is available in the Additional file 1).

Mass balance (%-recovery) of compounds were determined for the two experimental setups (Uni-L and Bi-L) using Eq. 6 and 7:

6 $$Recovery (\%), \,Uni-L= \frac{\left[\left({V}_{r}\times {C}_{r}\right)+\left({V}_{d}\times {C}_{d}\right)+\left({V}_{S}\times {C}_{S,\,apical}\right)+\left({V}_{S}\times {C}_{S,\,basolateral}\right)\right]}{\left({V}_{d}\times {C}_{0}\right)}\times 100$$

7 $$Recovery (\%), \,Bi-L= \frac{\left[\left({V}_{r}\times {C}_{r}\right)+\left({V}_{d}\times {C}_{d}\right)+\left({V}_{S}\times {C}_{S,\,apical}\right)+\left({V}_{S}\times {C}_{S,\,basolateral}\right)\right]}{\left({V}_{d,\,apical}\times {C}_{0,\,apical}\right) + \left({V}_{d,\,basolateral}\times {C}_{0,\,basolateral}\right)}\times 100$$

V_r_ and V_d_ represent volumes in receiver and donor chambers, respectively (500 μL apical and 1000 μL basolateral); V_S_ denotes the volume of sample taken (40 μL apical and 80 μL basolateral); C_0_ is the initial concentration in the donor chamber; while C_d_ and C_r_ represent final concentrations in donor and receiver chambers, respectively; C_S,apical_ and C_S,basolateral_ represent test compound concentrations before ending the experiment. In Eq. 7 (recovery in experiment using Bi-L), the compound is added on both sides, which means that both V_d_ and C_0_ contributed to both the apical and basolateral sides.

In these experiments, removing the matrice and accurately accounting for compound associated with the filter and cell monolayer proved challenging. Consequently, this factor was not taken into consideration in the calculation, leading to an expected lower recovery compared to bidirectional transport experiment (P_app_-experiment). Consequently, a recovery ≥ 50% was deemed acceptable. All compounds showed ≥ 60% assay recoveries (see Additional file 1), except for doxepin, fluoxetine, Way-100635 and N-desmethylclozapine, which had recoveries around 50%, and propranolol and altanserin with low values around 20%.

The total in vitro brain-to-BSA concentration ratio following 29 h of incubation (in vitro K_p,brain_) was estimated from the concentrations determined in the two chambers in these studies. The free fractions (estimated from Eqs. 1 and 2), were used to determine in vitro K_,p,uu,brain_, employing Eqs. 8 and 9, as follows:

8 $${In\, vitro \,K}_{p,brain}=\frac{{C}_{total, brain}}{{C}_{total, BSA}}$$

9 $$In\, vitro \,{K}_{p,uu,brain}={K}_{p,brain \times }\frac{{f}_{u,brain}}{{f}_{u, BSA}}$$

The notation C_total,brain_ and C_total,BSA_ refer to the total quantity of a compound present in the respective chambers at the final time-point; K_p,brain_ is the brain-to-BSA ratio; f_u,brain_ and f_u,BSA_ reflect the compound free fraction in brain homogenate and BSA, respectively; In vitro K_p,uu,brain_ is the ratio of concentrations of unbound brain-to-unbound BSA. Compounds were classified as having restricted brain penetration with K_p,uu,brain_ < 0.3, partially restricted brain penetration with K_p,uu,brain_ between 0.3 and 0.7, and unrestricted brain penetration with K_p,uu,brain_ > 0.7.

### Analysis of compounds in biological matrices

Samples from f_u_-binding and in vitro equilibrium brain-to-plasma distribution experiments, were mixed with an equal volume of the opposite matrice to achieve a final ratio of plasma: buffer, brain homogenate: buffer and brain homogenate: BSA of 1:1 (v/v). The calibration standards were matched in a similar way to cover a final concentration range of 1 to 1000 nM (1, 2.5, 10, 50, 200, 500, 1000 nM) for each compound plus three QC’s (10, 100, 800 nM). Samples, calibration standards and QC’s were extracted using acetonitrile containing appropriate bioanalytical internal standards then centrifuged (20 min, 3200 *g*, 4 ℃). The supernatants were diluted with an appropriate volume of water prior to analysis.

The bidirectional transport samples (P_app_-experiment) were processed in the same way as described above, but without use of calibration standards. Consequently, relative concentrations were determined by comparing peak area ratios ((analyte peak area (counts) * sample dilution factor)/Internal standard peak area (counts)).

The separation and determination of each compound was carried out using ultra-performance liquid chromatography (Acquity UPLC system; Waters, Milford, MA) coupled with tandem mass spectrometry detection in positive-ion electrospray ionization mode (Waters Xevo TQS triple quadrupole mass spectrometer; Waters, Milford, MA, US and Sciex Q-Trap 6500 + triple quadrupole mass spectrometer; AB Sciex, Framingham, MA, US).

## Results

**Fig. 1:**
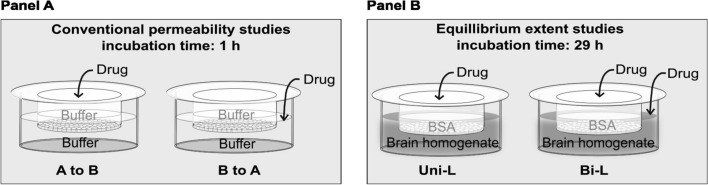
**A** The conventional permeability setup is shown, where discrete compounds were dosed in either the apical compartment or to the basolateral compartment, using transport buffer as matrice and a short incubation time of 1 h. **B** The in vitro equilibrium drug extent setup investigated in this publication, using BSA and brain homogenate and an incubation time of 29 h. Two different setups were investigated, either cassetted drugs were loaded to the apical chamber (into BSA, like a standard in vivo experiment) called the Uni-L method or they were added in both chambers called the Bi-L method

The aim of the study was to investigate whether a novel equilibrium in vitro method for estimating brain-to-plasma distribution can predict in vivo brain distribution (k_p,brain_ and k_p,uu,brain_) (as depicted in Fig. 1B). The subsequent sections will present data obtained from the conventional permeability method, along with estimates of protein media free fractions.

### Permeability and efflux ratio’s for selected compounds, obtained from conventional bidirectional transport studies in MDCKII-MDR1 cells

Conventional bidirectional 1-h transport experiments were performed in transport buffer using monolayers of MDCKII-MDR1 cells for 16 selected compounds, as described in the “Methods” section. The purpose of this series of experiments was to determine fluxes and permeabilities through bi-directional transport experiments, enabling comparison with similar studies in literature, and to rank the substrates in terms of P-gp interaction, using the ER. Table 1 summarizes mean in vitro permeability data and ER’s calculated from the transport data.

**Table 1:** In vitro bidirectional transport of 16 reference compounds across MDCKII-MDR1

Compound	Permeability (P_app_) (10^–6^ cm/s)		Efflux ratio	Human P-gp substrate classification	Permeability classification
	P_app,AtoB_	P_app,BtoA_			P_app,AtoB_
**Altanserin**	3.0 ± 0.6	3.4 ± 0.3	1.1	Unlikely	Moderate
**Antipyrine**	39 ± 1	48 ± 3	1.2	Unlikely	High
**Atenolol**	< LLOQ	< LLOQ	NA	NA	Low
**Buspirone**	21 ± 2	20 ± 3	0.9	Unlikely	High
**Cimetidine**	< LLOQ	2.5 ± 0.2	NA	Possible	Low
**Citalopram**	4.7 ± 0.4	7.7 ± 0.3	1.7	Possible	Moderate
**Diphenhydramine**	9.8 ± 1.3	16 ± 1	1.6	Possible	Moderate
**Doxepin**	2.0 ± 0.1	2.2 ± 0.1	1.1	Unlikely	Moderate
**Fluoxetine**	0.4 ± 0.1	0.4 ± 0.1	1.0	Unlikely	Low
**Gabapentin**	2.9 ± 0.7	1.6 ± 0.2	0.5	Unlikely	Moderate
**Indomethacin**	3.7 ± 0.5	2.7 ± 0.3	0.7	Unlikely	Moderate
**Metoclopramide**	24 ± 1	49 ± 3	2.0	Likely	High
N-desmethylclozapine*	2.1 ± 0.5	4.4 ± 0.3	2.1	Likely	Moderate
**Propranolol**	4.5 ± 0.4	3.7 ± 0.1	0.8	Unlikely	Moderate
**Risperidone**	8.5 ± 1.2	37 ± 4	4.4	Likely	Moderate
**Way-100635**	13 ± 1	17 ± 3	1.3	Unlikely	High

MDCKII-cells transfected with the human P-gp-transporter (hMDR1). Data represents mean ± SD of one test occasion of three individual filters, ‘n’ denotes test occasions, and ‘total N’ denotes the total number of replicates (n = 1, total N = 3). Recovery > 75% in total recovery (with monolayer accounted for)

^*^ Also tested with an inhibitor present given P_app (A-B)_: 3.6 ± 0.2 and P_app (B-A)_: 2.9 ± 0.5, with an ER of 0.8

Permeability classification: low: P_app_ < 1, moderate > 1 P_app_ < 10, and high: Papp > 10

P-gp substrate classification: likely P-gp substrates: ER > 2, possible substrates: ER > 1.5, and unlikely substrates: ER < 1.5

*NA* not available

Permeability and ER’s were calculated for 14 compounds, while for atenolol and cimetidine, the observed fluxes were too small to be quantified (< LLOQ) in either one or both directions. Compounds included in the transport experiments ranged from low, moderate, and highly permeable compounds. Antipyrine and fluoxetine exhibited the highest and lowest detectable P_app,AtoB_ values, which were 39 × 10^–6^ cm/s and 0.4 × 10^–6^ cm/s, respectively. Among the included compounds, 11 were classified as unlikely human P-gp substrates (ER < 1.5) and 3 as likely substrates (ER > 2). The highest ER’s were found for risperidone (ER: 4.4). Diphenhydramine and citalopram exhibited ER’s of 1.6 and 1.7, respectively, which suggests they may be potential P-gp substrates. Cimetidine was also considered a possible substrate as its permeability in the AtoB direction was < LLOQ, but detectable in the BtoA direction.

### BSA at a concentration of 700 μM effectively mimics in vivo binding

**Fig. 2:**
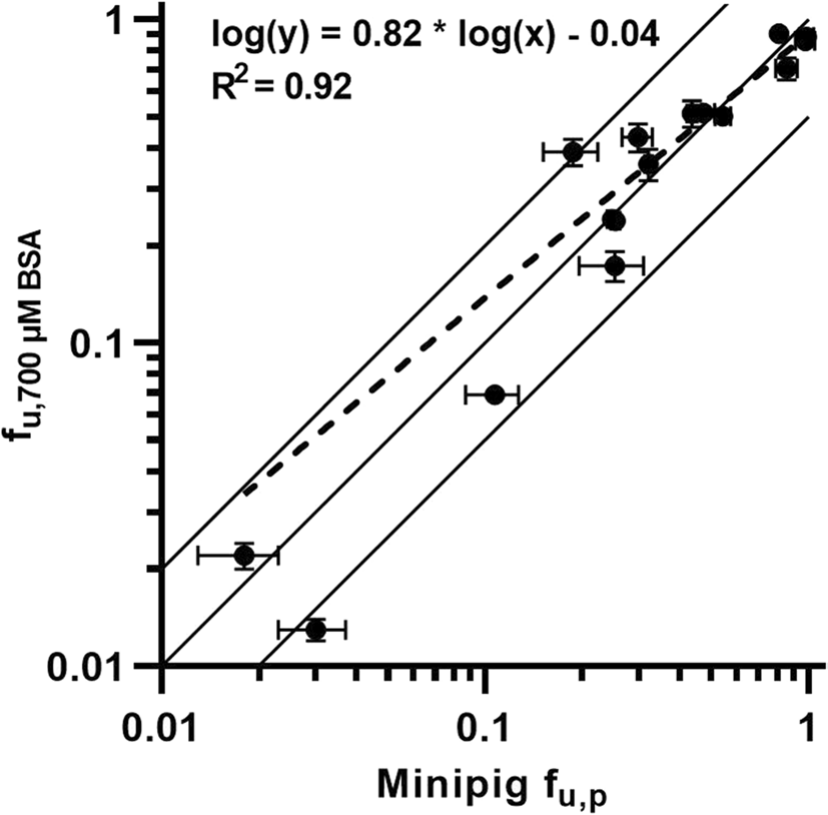
Comparison of log-transformed 700 μM BSA free fractions (f_u, 700 μM BSA_) and Göttingen minipig 100% plasma (f_u,p_). The solid lines represent the line of unity and twofold difference, while the dotted line shows the line of best fit from linear regression analysis. Mean data are presented (n = 16, triplicate determinations on a single test occasion for all compounds, except for Altanserin and Way-100635, which were tested on two occasions). The data and standard deviations (SD) are shown as mean and variation between all 6 replicates)

The next step, in order to mimic a more in vivo like setup, was to design the composition of protein-containing culture media to be used in the apical compartment (blood side) for the long-term equilibrium-distribution experiments. Initially, diluted plasma was used in the apical (blood-side) compartment as described in “Methods”. However, this disrupted cell monolayer integrity and proved to be physically unstable. Consequently, the plasma was replaced with a simpler matrice consisting of conventional cell media and BSA. Figure 2 shows a comparison of f_u_ values of the compounds in 700 μM BSA in phosphate buffer and Göttingen minipig plasma.

According to Christoffersen et al. a BSA concentration of 700 μM (equivalent to 46.5 *g*/L of albumin), was determined in the plasma of female Göttingen minipigs at 24 weeks of age [24].

The measured f_u, 700 μM BSA_ for the reference set of compounds ranged 70-fold, from highly bound to highly unbound (0.013 to 0.904 for indomethacin and metoclopramide, respectively). The free fractions for the 16 reference compounds in BSA were similar and highly correlated with previously determined values [20] in Göttingen minipig plasma (R^2^ > 0.9, regression slope: 0.82, Fig. 2; with most values clustering close to the unity line. The exceptions being N-desmethylclozapine and indomethacin with values greater than twofold different.

These data supported that BSA could act as a viable plasma substitute in the apical (blood-side) compartment and circumvent the cell viability issues associated with using whole plasma. For the purpose of preserving the protein ratios (with the brain necessitating a 20-fold dilution), the incubations involved a 20-fold dilution of BSA as well.

### Comparing predicted and experimentally determined free fraction in vitro: evaluating the impact of 35 μM BSA and a 20-fold dilution of brain homogenate

In order to calculate in vitro K_p,uu,brain_, it was necessary to estimate the free fraction, f_u_, of the compounds under the relevant assay conditions, i.e. 35 μM BSA on the apical side of the cell monolayer and 20-fold diluted rat brain homogenate on the basolateral side.

Experimentally determined free fraction data (using Eq. 1) and predicted data (using Eq. 2) for the 16 reference compounds are shown in Table 2. The experimental data revealed that for 11 out of 16 compounds, the difference between the two methods was less than 10%, indicating good agreement between methods. However, for three compounds (antipyrine, diphenhydramine, indomethacin), a difference, ranging from 10 to 20% in the brain free fraction data was observed, while another three compounds (altanserin, doxepin, indomethacin) showed a difference exceeding 20%. It is worth noting that the greatest differences were observed for altanserin and indomethacin in the BSA-medium (with a difference ~ twofold).

**Table 2:** Free fraction (f_u_) determined in vitro and predicted using Eq. 2

Compound	Determined f_u_ (%)		Predicted f_u_ (%) *	
	Brain (D = 20)	BSA 35 μM (D = 20)	Brain (D = 20)	BSA 35 μM (D = 20)
Altanserin	22.9 ± 1.4	45.1 ± 3.5	22.1	26.8
Antipyrine	84.3 ± 4.2	> 100	> 100	99.9
Atenolol	95.2 ± 2.1	> 100	> 100	99.9
Buspirone	83.6 ± 9.5	84.2 ± 2.9	83.1	90.4
Cimetidine	93.8 ± 5.0	> 100	95.6	99.2
Citalopram	51.3 ± 6.6	97.3 ± 2.7	47.9	96.0
Diphenhydramine	62.6 ± 6.1	> 100	72.8	94.7
Doxepin	24.8 ± 5.0	92.2 ± 2.9	33.0	87.1
Fluoxetine	5.2 ± 0.5	66.0 ± 3.2	5.7	70.6
Gabapentin	94.6 ± 1.5	98.4 ± 6.2	93.4	99.8
Indomethacin	35.8 ± 8.0	19.6 ± 1.2	42.8	38.2
Metoclopramide	87.8 ± 3.8	> 100	90.7	98.8
N-desmethylclozapine	13.1 ± 0.6	89.2 ± 2.9	13.9	82.1
Propranolol	35.0 ± 1.2	89.7 ± 1.8	37.4	86.8
Risperidone	74.5 ± 1.9	91.8 ± 1.6	69.9	89.4
Way-100635	74.8 ± 8.0	80.2 ± 0.9	71.8	87.1

Data represents mean ± SD of one test occasion of three individual filters, ‘n’ denotes test occasions, and ‘total N’ denotes the total number of replicates (n = 1, total N = 3), and predicted f_u_ are shown as mean values

^*^ Calculated with use of Eq. 2, see Sect. "In vitro BSA and brain homogenate binding for reference compounds"

*D* denotes the dilution factor of the matrix

### Attaining drug equilibrium via MDCKII-MDR1 cells

Using the compound in vitro unbound fractions, the compound unbound concentrations at equilibrium were then estimated in the cell experiments. Compounds were added either to the BSA side (Uni-L) or to both sides (Bi-L) and allowed to equilibrate for 29 h as described in the “method” section.

Barrier integrity was evaluated by measuring the TEER before and after exposure. Through the experiments, a 25 ± 16% (total N = 90) decrease in average TEER over 29 h of incubation was observed (see Additional file 1). In this way, the barrier properties of the MDCKII-MDR1 cell monolayers maintained an average TEER value of 137 ± 13 Ω *cm^2^ (total N = 90) at the end of incubation. The pH in the brain homogenate was observed to shift from 7.5 to 8 during the 29-h incubation. A similar trend towards higher pH was noticed in the BSA compartment, but to a lesser extent.

**Fig. 3:**
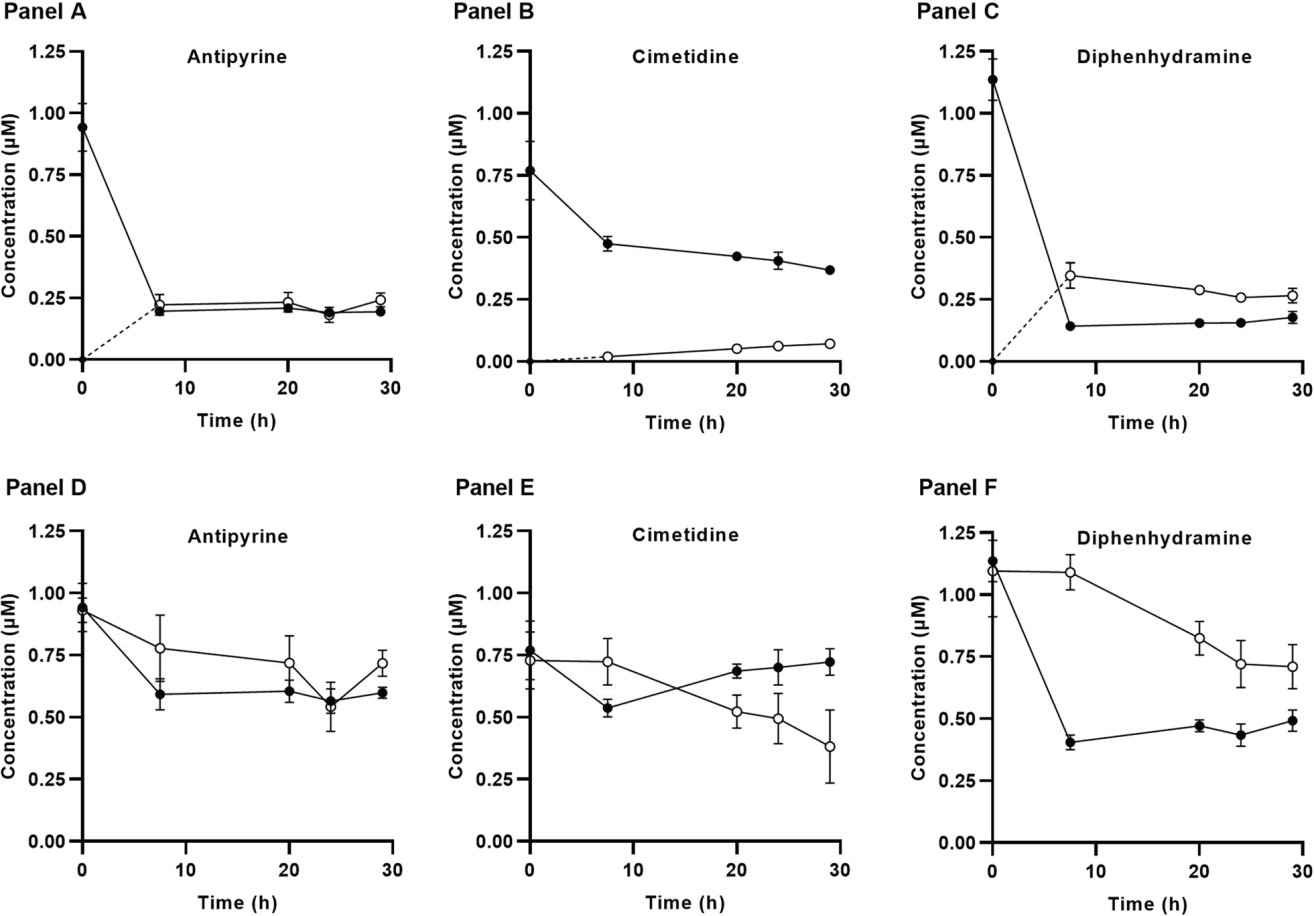
The concentration–time profiles for three exemplified compounds using Uni-L (loading compound apically) in **A**–**C** and Bi-L (loading compound into both compartments) in **D**–**F**. Antipyrine is a passive permeable compound, cimetidine is a P-gp substrate, and diphenhydramine is a potential uptake substrate. Concentrations in the apical (black circles) and basolateral compartments (open circles) of the in vitro K_p,uu,brain_ experiment are shown, compounds were loaded at nominal concentrations of 1 μM. Data represents mean ± SD of three occasion/cell passages of three individual filters, ‘n’ denotes test occasions, and ‘total N’ denotes the total number of replicates (n=3, total N=9)

During the incubation period, the concentration of 11 out of 16 compounds reached equilibrium between the “plasma” and “brain” compartment with Uni-L, (representative compounds presented in Fig. 3A–C, while the profiles of all compounds are available in the Additional file 1). For atenolol, gabapentin and cimetidine, which exhibited low permeability, equilibrium conditions were not fully achieved (see cimetidine data presented in Fig. 3B and E). Using the Bi-L method similar trends between brain/plasma distributions were observed, though with a higher degree of variation in the plots (Fig. 3D–F).

### Comparison of in vitro and in vivo determined brain distribution (K_p,brain_ and K_p,uu,brain_) parameters

The in vitro K_p,brain_ values were obtained from the ratio of drug-compound on the brain and blood side respectively, at equilibrium. In vitro K_p,uu,brain_ values were obtained by transforming K_p,brain_ values using the obtained f_u_ values, as described in the “Method” section.

**Fig. 4:**
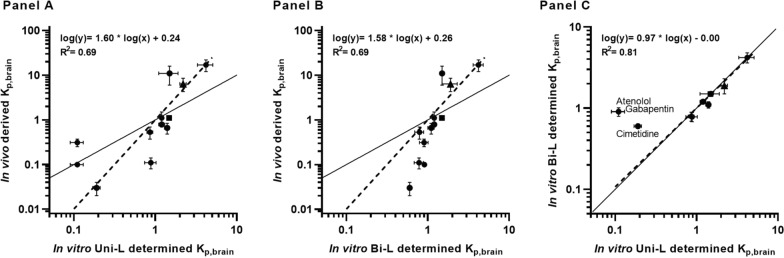
Correlation between log-transformed in vitro determined K_p,brain_ and in vivo derived K_p,brain_ for selected reference compounds (n = 12), excluding uptake substrates (metoclopramide, doxepin, diphenhydramine, and fluoxetine). **A** depicts Uni-L, while **B** represents Bi-L. The in vitro data represents the mean ± SD of three passages conducted on three individual filters (n = 3, total N = 9), while the in vivo data represents three minipigs (n = 1, total N = 3). ‘n’ denotes test occasions, and ‘total N’ denotes the total number of replicates. **C** presents a comparison between the two applied in vitro methods. Low recovery compounds are shown in distinct symbols, altanserin as a square, and propranolol as a triangle

Comparing in vitro K_p,brain_ values determined in the present study to previously reported in vivo determined K_p,brain_ values [20] (see Additional file 1 for tabulated data), a correlation (R^2^ = 0.69) was obtained between in vitro and in vivo values of K_p,brain_ for both Uni-L and Bi-L (Fig. 4A and B). It is also worth noting that slopes of similar magnitude at approximately 1.6 were observed for both methods, which indicates a similar in vivo predictivity. The range of in vitro K_p,brain_ values was between 0.1 to 4.2, while the in vivo K_p,brain_ values ranged from 0.1 to 17. Compounds with low in vitro permeability, P_app_ < 1 (atenolol and cimetidine, and the moderate permeable compound gabapentin) exhibited significant differences between the two methods, although the results were otherwise comparable (Slope: 0.97, R^2^ = 0.81) (Fig. 4C). For the correlation in Fig. 4, uptake transporter substrates were excluded (since the MDCKII-MDR1 cell line has not demonstrated the presence of, for instance, organic cation transporters (OCTs)).

**Fig. 5:**
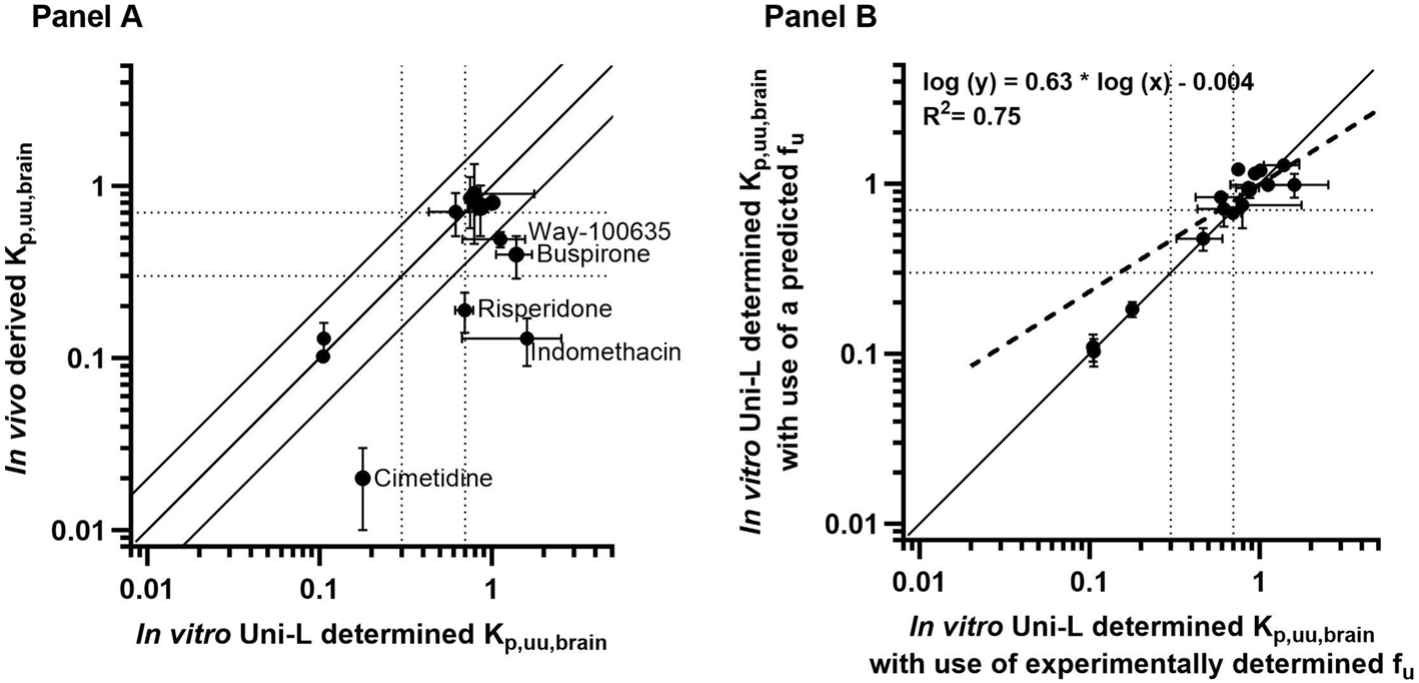
**A** depicts the relationship between log-transformed in vitro and in vivo derived K_p,uu,brain_ values for selected reference compounds (n = 12) using the Uni-L setup. **B** compares K_p,uu,brain_ values using predicted and in vitro determined free fractions in the experimental matrices. The in vitro data represents the mean ± SD of three passages conducted on three individual filters (n = 3, total N = 9), while the in vivo data represents three minipigs (n = 1, total N = 3). ‘n’ denotes test occasions, and ‘total N’ denotes the total number of replicates. The solid lines represent the line of unity and a twofold difference, and the dotted lines represent different levels of brain penetration: restricted (K_p,uu,brain_ < 0.3), partially restricted (K_p,uu,brain_ between 0.3 and 0.7), and unrestricted (K_p,uu,brain_ > 0.7)

Subsequently, K_p,uu,brain_ values were calculated using predicted and determined in vitro in BSA and rat brain homogenate f_u_ at relevant matrice-protein concentrations (Fig. 5A and B).

In vivo and in vitro K_p,uu,brain_ values for the reference subset (12 compounds) did not show a significant correlation. It is however noteworthy that 58% (7 out of 12) of the Uni-L determined K_p,uu,brain_ values fell within a twofold range of the in vivo derived values (Fig. 5A). Furthermore, 67% (8 out of 12) of the compounds that underwent in vitro Uni-L testing were correctly classified according to the in vivo brain penetration categories e.g. restricted (K_p,uu,brain_ < 0.3), partially restricted (K_p,uu,brain_ between 0.3 and 0.7), and unrestricted (K_p,uu,brain_ > 0.7). However, it is essential to highlight that the in vitro classification for certain compounds, buspirone, indomethacin, risperidone, and Way-100635 differed to their in vivo classification. A notable similarity was identified when comparing the K_p,uu,brain_ estimates calculated from predicted and in vitro determined free fractions (Fig. 5b).

## Discussion

Estimates of drug transport from blood to brain parenchyma are essential in CNS drug development. At present, available techniques and methods range from relatively simple and inexpensive cell culture permeation studies to costly in vivo PK studies where the distribution of a drug compound between brain parenchyma and plasma (K_p,brain_) can be measured [25, 26]. The free concentrations in brain and plasma can be estimated in vitro or measured in vivo, and the ratio between unbound drug in brain and plasma can be calculated, providing the parameter K_p,uu,brain_ [27]. K_p,uu,brain_ is an estimate of actual extent of brain penetration, as compared to the more mechanistic permeability values and ER’s obtained from flux experiments in in vitro cell culture setups. Although K_p,uu,brain_ estimates are considered the gold standard for investigating brain drug disposition, the experimental work can be costly and time consuming, and the throughput is considered to be a limiting factor in CNS drug development [28].

In the present study, we investigated the feasibility of refining in vitro transport studies using the P-gp expressing MDCKII-MDR1 cell line to estimate long term distribution between the apical “blood” and the basolateral “brain” compartment, in order to obtain in vitro K_p,brain_ and K_p,uu,brain_ values in a setup with apical and basolateral solutions with high protein content mimicking physiological ratio’s.

We observed a correlation between in vitro and in vivo K_p,brain_ values for the reference set of compounds (Fig. 4). The in vitro K_p,uu,brain_ was calculated from K_p,brain_ using Uni-L, as this method is comparable to the in vivo method (where compounds are dosed in blood corresponding to the in vitro apical solution with BSA). We could not obtain a correlation for K_p,uu,brain_ as shown in Fig. 5A. However, the in vitro method accurately predicted the K_p,uu,brain_ classification of compounds, as evidenced in Table 3. The table also provides a comparison by capturing the more conventional estimated ER values.

**Table 3:**
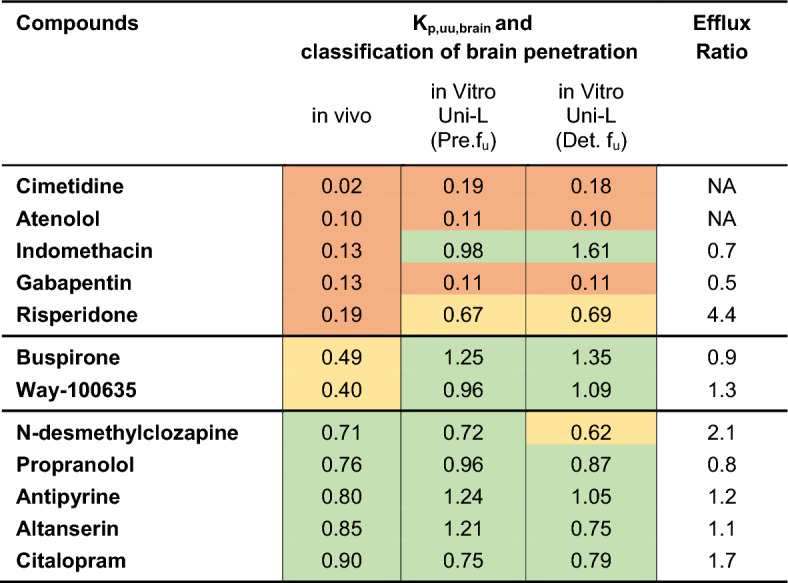
In vivo Göttingen minipig K_p,uu,brain_ (from previous publication [20],) and in vitro K_p,uu,brain_ from Uni-L calculated with use of predicted (Pre.f_u_) and determined (Det.f_u_) fu data

The brain penetration classification is shown in three colours for compounds (n = 12): restricted (K_p,uu,brain_ < 0.3) in orange, partially restricted (K_p,uu,brain_ between 0.3 and 0.7) in yellow, and unrestricted (K_p,uu,brain_ > 0.7) in green. In a separate column is the Efflux ratio demonstrated from a conventional transport study (also captured in Table 1). Compounds considered to be uptake substrates have been excluded (metoclopramide, doxepin, diphenhydramine, and fluoxetine)

Table 3 provides evidence for successful classification of drug compounds classified as having unrestricted brain penetration in vivo (compounds with K_p,uu,brain_ > 0.7) using the Uni-L in vitro K_p,uu,brain_ setup as 5 out of 5 compounds were correctly classified using predicted f_u_ values and 4 out of 5 compounds using measured f_u_ values. The Uni-L in vitro setup is, however, challenged by the partially restricted compounds (K_p,uu,brain_ between 0.3 and 0.7). Buspirone and Way-100635, fall in this category according to their in vivo data but exhibited unrestricted brain penetration based on results from the in vitro setup. The in vitro model demonstrated a good level of predictability for restricted compounds (compounds with K_p,uu,brain_ < 0.3) as 3 out of 5 compounds were correctly classified, with a striking similarity between the in vitro and in vivo K_p,uu,brain_ values for gabapentin and atenolol (Table 3). The two likely P-gp substrates, risperidone (ER = 4.4) and N-desmethylclozapine (ER = 2.1), exhibited distinct BBB classification. Risperidone was categorized as a restricted compound, while N-desmethylclozapine was identified as an unrestricted compound.

For indomethacin, risperidone, buspirone, and Way-100635 the similarity between in vitro and in vivo K_p,uu,brain_ values were less pronounced. Indomethacin exhibited in vivo and in vitro K_p,uu,brain_ values of 0.13 and ≥ 1, respectively. Indomethacin has been reported to be a substrate for the organic anion transporter 1 (OAT1) [29–31] and organic anion transporter 3 (OAT3) [30]. These transporters are primarily expressed in the basolateral (blood-side) membrane of proximal tubule cells in the kidney [32, 33], and only indistinct expression of OAT1 have been detected in the brain (OAT3 was not investigated in the Hosoyamade et al. study [32]). Considering the expression levels of these transporters, it is worth noting that the MDCKII cell line used in the present study is isolated from a canine Cocker Spaniel Kidney [34].This fact could potentially explain why indomethacin gave a higher in vitro K_p,uu,brain_ value, given the presence of transporters that facilitate its transport in kidney cells. Similarly, the in vitro K_p,uu,brain_ value for Way-100635 was higher than its in vivo value (K_p,uu,brain_ ~ 1 and 0.40, respectively). Interestingly, Liu H et al. report this compound to be a substrate for rodent P-gp but not to interact with human P-gp [5, 35, 36]. This could aid in reconciling the observations made in our current study involving MDCKII monolayers expressing human P-gp.

Based on pKa values for selected compounds (see Additional file 1), it is possible that altanserin, buspirone, and risperidone, with QSAR (Simulation-Plus ADMET^™^ Predictor software version 10.3) predicted pKa values of 7.4, 7.2, and 8.0 respectively, could have been influenced, in terms of their degree of ionization and permeation rate, by the observed minor pH changes during the 29 h of incubation (the pH shifted from 7.4 to 8 during the 29 h of incubation). Altanserin was unfortunately one of the compounds with low recovery (~ 20%). In contrast, both buspirone and risperidone exhibited differences between their in vitro and in vivo K_p,uu,brain_ results, and it may be that the increased in vitro pH decreased their extent of ionization in turn enhancing their in vitro membrane permeation. Using the Uni-L method, time-concentration profiles show that equilibrium was achieved in both the “plasma” and the “brain” compartments, while with the Bi-L method, the concentration in the brain chamber did not reach equilibrium during the incubation time (See concentration–time profiles in Additional file 1). This precisely highlights the observed trend between the two matrices after 29 h of incubation, where the brain compartment shifted from a pH of 7.5 to 8, while the pH change in the BSA compartment was less pronounced. When a compound's pKa value is in the range of a pH change, this effect becomes more significant. To address this issue, shortening the incubation time for compounds with such physicochemical characteristics could be beneficial. In scenarios like this, both in vitro methods (Uni-L and Bi-L) contribute valuable insights and aid in the validation of the estimated results.

Furthermore, when dealing with compounds of low permeability, the in vitro setup of Bi-L method could offer a more precise representation of the equilibrium situation. This is in contrast to the Uni-L method, in which reaching equilibrium using cell systems might not be feasible within an acceptable incubation time.

The present study demonstrated a correlation among compounds, but without involving those with affinity for uptake transporters. This is a limitation of both the study and the MDCKII-MDR1 line as a screening tool. However, as most registered CNS drug compounds are small and lipophilic, this is generally not perceived as a major limitation of the cell line but should be kept in mind in development programs where transporters are specifically targeted.

The in vitro Uni-L method demonstrated effective prediction of in vivo brain penetration for the majority of reference compounds. However, the lack of direct translation of in vitro K_p,brain_ values to in vivo K_p,brain_ is not completely understood from our data. In our study, we identified a correlation between in vivo and in vitro* K*_*p,brain*_, but the slope differed from 1 (Fig. 4A, B, slopes of 1.6 observed for both Uni-L and Bi-L) suggesting the dynamic range in vitro was significantly lower than in vivo. One possible reason for this observation could be due to a disparity between the surface area available for flux in the in vitro setting (1.12 cm^2^) versus the large surface area in the brain capillaries. This implies that there exists a ratio between the capillary surface area and capillary plasma volume versus the insert area and apical/basolateral buffer volume. Notably, the in vitro and in vivo data correlate well for K_p,brain_ values above 0.3. The most significant disparity, which arose with very low permeable compounds (e.g., cimetidine and atenolol), can be attributed to the challenge of fully attaining equilibrium in both in vitro methods. This is possibly due to the interaction of these compounds with the limited cell surface area in vitro. As a consequence, the in vitro and in vivo K_p,brain_ data do not demonstrate a slope of 1 (as shown in Fig. 4A, B).

However, it is also essential to consider that in vivo determination of K_p,brain_ for low permeable compound poses a challenge. Small changes in measured in vivo steady-state plasma concentrations could be masked by bioanalytical variability. The use of an in vivo method involving administration of an intravenous bolus and intravenous infusion might lead to the appearance of steady state in plasma concentrations. However, steady-state conditions may not have been reached in brain, resulting in a lower K_p,brain_ value. Consequently, the observed disparity in cimetidine and atenolol could be attributed to a bias towards non-equilibrium in vivo conditions in the brain caused by the commonly employed in vivo method combined with a challenging low permeability, as previously demonstrated in rats by Chen et al. [37].

The use of the two different f_u_ methods had minimal impact on the K_p,uu,brain_ rank order for most compounds, as shown in Table 3 (except for N-desmethylclozapine). Given these data, it may be possible in the future to triage compounds in the initial screening phase, using the in vitro K_p,uu,brain_ experiment setup before proceeding to more expensive in vivo K_p,uu,brain_ experiment. The use of in vitro cell culture data to estimate brain drug disposition has also been addressed in other studies. Recently Nikolai et al. and Storelli et al. have effectively predicted human K_p,uu,brain_ using PBPK models coupled with inputs from conventional in vitro permeability assays, and transporter proteomics [38, 14]. However, only few studies have attempted to create an in vitro setup that enables estimation of drug distribution parameters such as K_p,brain_ and K_p,uu,brain_. In 2012, Culot et al. introduced a technique to generate the K_p,uu,brain_ parameter within a single in vitro experiment. They achieved this by utilizing a co-culture cell system that included both endothelial and glial cells. The presence of glia cells aimed to replicate the non-specific binding of drug compounds in the brain. Within this system, a 1-h incubation period was used to estimate and establish equilibrium concentrations. The investigation revealed that 87% of the predicted in vitro equilibrium K_p,uu,brain_ values were within a twofold range of the corresponding in vivo values [16]. While Culot et al. took an initial step towards replicating in vitro K_p,uu,brain_, they did not adequately account for equilibrium conditions. Our research has expanded upon this by achieving improved equilibrium conditions using well-tolerated protein on both sides of the cell system. This offers a wide range of possibilities for exploring different transporter systems and their influence on the extent of drug distribution in the brain across various cell systems.

Our model adheres to the principles of the 3R’s (Reduce, Refine, Replace), benefiting both scientific advancement and animal welfare [6–9]. By reducing the reliance on animal models, our approach exemplifies a more ethical path. One of the key advantages of this method is its cost-effectiveness, enhancing research efficiency and accelerating the pace of brain drug distribution in drug discovery. However, a drawback of the current method is the lack of uptake transporters in the MDCKII-MDR1 cell line. However, this is unlikely to be a concern in research screening programs where passive permeation rate and efflux transporter classification remain the focus. In the future, the current model could be adjusted to study various transporter systems or their combinations, enhancing our understanding of drug disposition under these conditions. Additionally, the model could be made more translatable by using cell lines that are more physiologically relevant to the expected in vivo model.

## Conclusion

In the present study we investigate a novel in vitro setup using P-gp expressing MDCKII-MDR1 cells and high protein bathing solution for determining brain drug distribution parameters K_p,brain_ and K_p,uu,brain_.

We observed a good correlation between in vitro and in vivo K_p,brain_ values (R^2^ = 0.69, Slope: 1.6), highlighting the in vitro model’s potential to predict in vivo drug brain penetration. The ‘Uni-L’ in vitro setup correctly classified 5 out of 5 unrestricted compounds and 3 out of 5 restricted compounds. This correlation is attributed to the use of brain homogenate and BSA, replicating a more physiologically relevant, in vivo-like protein environment on both sides of the cell monolayer at equilibrium. The setup effectively categorizes brain penetration of the majority of reference compounds, successfully predicting 8 out of 12 compounds (with potential reasons for the prediction failure of 4 compounds). With minor refinements, this model could be evolved into a more dependable in vitro tool for predicting brain penetration of novel research and development compounds. As such, it holds the potential for early-stage screening, reducing the need for in vivo experiments, thereby enhancing CNS drug discovery efficiency.

### Supplementary Information

**Additional file 1.** Supplemental figures and tables.

## Data Availability

The dataset supporting the conclusions of this article is included within the article and its additional file (Additional file 1).

## Abbreviations

BSA: Bovine serum albumin
Bi-L: Bilateral
CNS: Central nervous system
Det.f_u_: Determined free fraction
f_u,__b_: The unbound fraction in brain homogenate
f_u,__p_: The unbound fraction in plasma
K_p__,brain_: Total brain-to-plasma ratio
K_p__,uu,brain_: Unbound brain-to-unbound plasma ratio
LC: Liquid chromatography
MS: Mass spectrometry
P-gp: P-glycoprotein
Pre.f_u_: Predicted free fraction
SD: Standard deviation
TEER: Transendothelial electrical resistance
Uni-L: Unilateral
